# Supplementary material for: Prevalence and determinants of poor glycemic control among diabetic chronic kidney disease patients on maintenance hemodialysis in Tanzania
Source: PLoS One. 2025 Feb 10;20(2):e0306357. doi: 10.1371/journal.pone.0306357 (PMC11809847; doi:10.1371/journal.pone.0306357)
Supplement: Attachment — Submitted filename: matrix_response_for Manuscript.docx [file pone.0306357.s003.docx]

**RESPONSE REVIEWER COMMENTS**

**Title: Prevalence and determinants of poor glycemic control among diabetic chronic kidney disease patients on maintanance hemodialysis in Tanzania**

| **S/N** | **Reviewer** | **Comments from the reviewer** | **PI’s response** | **Line Number**  **where changes have been made** |
| --- | --- | --- | --- | --- |
| **1** | **Editor comment** | Sample size calculation In this section you mentioned the sample size is 380 but in the result you said n=233. In the table 3 you mentioned the sample is 170. Please mention the chart flow of this study in the method section including the sample that you have used | A sample size of 380 participants was obtained using a prevalence of 54% poor glycemic control in diabetic patients on hemodialysis conducted in Canada with a 95% confidence level and a margin of error of 5% (13). However, due to a restricted number of diabetic hemodialysis patients at dialysis centers, the sample size was recalculate for a finite population using the estimated diabetic-CKD population at thirteen (13) data collection sites, which was 600. The recalculation yielding a sample size of 238 people whereby 5 patients were excluded from the study due to abnormal records of HbA1c due to incorrect documentation. Therefore only 233 patients were included in the final analysis. | **64-72** |
| **2** | **Reviewer #1** | This manuscript describes the Prevalence and determinants of poor glycemic control among diabetic chronic kidney disease patients on maintenance hemodialysis in Tanzania. This article is fairly well written but could use one more review by a native English speaker as there is unnecessary addition of commas in several areas and word "the" appears to be missing in several areas. In addition, some of the sentence are a little confusing and seem like the words were mixed up. | Paraphrasing to remove unnecessary coma and mixing of sentences has been done throughout the manuscript. |  |
|  |  | The range of HBA1c set by the author is unclear to me. There are a lot of papers that consider HBA1c of <6 % to be normal. The Lancet laboratories Tanzania consider the HBA1C less than 5.7% to be normal. HBA1c level of less than 5% is considered risk for diabetes patients. <https://www.cerbalancetafrica.co.tz/media/5kih0nky/diabetes-panel-tanzania.pdf> Overall, these is a very good start here. In order for this to be a valuable contribution to published literature, I would encourage the authors to take a few more steps to increase the impact of their research. | For diabetic patient without CKD, the range of HbA1c <5.7 Might be normal, but for Diabetic CKD patient based on Tanzania dialysis guidelines, KDIGO clinical practice guidelines for diabetic management in CKD (2022) and epidemiological data, an ideal HbA1c between 6% and 8%, for Diabetic CKD patients on maintenance hemodialysis. This is due to altered glucose homeostasis, inaccuracy of glycemic regulation metrics, and changed renal pharmacokinetics of anti-hyperglycemic agents (AHA) due to renal dysfunction, uremic milieu, and dialysis therapy | **24-30** |
|  |  | **Abstract:** Minimize the use of commas in the abstract and make sentences easier to grasp | Paraphrasing by minimizing the use of commas has been done. | **Page 2** |
|  |  | **Results** Please rewrite the line “While the predictor of HbA1c <6% was the type of dialyzer used (0.57 (95% CI 0.36 – 0.87) p = 0.020)”. And information regarding HBA1c <6 is not sufficient | Reviewed to  “While lower risk of HbA1c <6% was observed among patients dialyzed using glucose containing dialyzer 0.57 (95% CI 0.36–0.87) p = 0.020.” | **Page 2** |
|  |  | Line 104- 88.4 should be written as 88.4% | Revised to “The majority (93.6%) of participants had CKD stage five, **88.4%** were on dialysis three times per week, and 77.2% (n = 202) had attained dialysis adequacy” | **116** |
|  |  | Line 22- Hyper dislipidemia should be written either dyslipidemia or hyperlipidemia.  Line 26-28- Please reconsider writing these lines as these lines are difficult to understand. Please verify either these lines are according to WHO guidelines for Diabetes Mellitus or not | Revised to dyslipidemia  Revised to “The Tanzania guideline on hemodialysis recommends all patients have baseline measurements of their blood sugar level and glycated hemoglobin (HbA1C) at the time of initiation of dialysis therapy and recommends HbA1c between 7% and 8% as an ideal target (9). Furthermore, KDIGO clinical practice guidelines for diabetic management in CKD (2022) recommend an ideal HbA1c between 6% and 8% for diabetic CKD patients on maintenance hemodialysis (10). This is also supported by the previous epidemiological study, which reported that HbA1c from 6% to 8%, is associated with decreased mortality rates.” | **22**  **24 - 30** |
|  | **Reviewer #2** | **Abstract** Clarity and Conciseness: The abstract effectively summarizes the study's background, methodology, results, and conclusions. It is concise and provides a good overview of the research. * Minor Typo: Change "was considered when glycated hemoglobin (HbA1c) levels were < 6% or >8%" to "were considered when glycated hemoglobin (HbA1c) levels were < 6% or >8%." | Paraphrased to  “Which were considered when glycated hemoglobin (HbA1c) levels were < 6% or >8%.” | **Page 2** |
|  |  | **Literature Review:**  The introduction references relevant studies and statistics, but could benefit from a more detailed discussion of the specific challenges faced in Tanzania compared to other regions. | We worked on literature review for this study, but studies regarding the prevalence and determinants of poor glycemic control among diabetic CKD patients on hemodialysis is quite limited in sub-Sahara Africa including Tanzania. Therefore, all available literature has been cited. |  |
|  |  | **Inclusion and Exclusion Criteria:**  The inclusion and exclusion criteria are appropriate. However, the exclusion criteria for patients without recent HbA1c results might have been further elaborated upon, considering potential biases. | **Further elaboration has been provided.**  **“Excluding patients without recent HbA1c results was essential to ensure data accuracy, consistency, and reliability while reducing the risk of biases such as misclassification, selection, and confounding.”** | **62 - 64** |
|  |  | **Sample Size Calculation:**  The sample size calculation is justified based on previous prevalence data. It would be helpful to mention any adjustments or considerations made during the study due to recruitment challenges. | Adjustments made due to challenges encoutered in enrolling patients have been described by recalculating using the finite population. | **66 - 74** |
|  |  | **Data Collection:**  The data collection process is well detailed. The use of both primary and secondary data is a strength, but more information on the validation of the semi-structured questionnaire could add to the credibility. | Additions made  “The tool was reviewed by experts on the subject to ensure relevance, clarity and comprehensiveness of the questions was assessed. Then the tool was pre-tested to a small group of diabetic CKD patients on hemodialysis whereby the results of the pretest was further used to refine the tool.” | **85 - 88** |
|  |  | **Statistical Analysis:**  The statistical methods used are appropriate. The use of univariate and multivariable regression models is well justified, but providing more details on how potential confounders were controlled in the analysis would be beneficial. | The paragraph has been modified by addition of how confounders were controlled.  “In a multivariable analysis, potential confounders were controlled by including them as covariates (independent variables) in the regression model. This statistical analysis isolated the effect of the independent variables such as age, dialysis duration, comorbidities etc. on the outcome of interest (glycemic control) while accounting for other factors that might influence the outcome. Variables with p<0.1 on univariate analysis were considered for multivariate regression. A p-value of <0.05 was considered statistically significant.” | 103 - 109 |
|  |  | **Interpretation of Findings:**  The discussion effectively interprets the results and places them in the context of existing literature. However, some parts are repetitive, particularly concerning the importance of glycemic control. | The discussion part has been revised to omit repetitions. | **Page 13 - 15** |
|  |  | **Strengths and Limitations:**  The discussion of the study's strengths and limitations is comprehensive. However, the potential impact of the exclusion criteria (patients without HbA1c results) on the findings could be more thoroughly examined. | Changes made  **“Excluding such patients could have resulted in selection bias, whereby patients without HbA1c results may systematically differ from those with recent results. However, for the purpose of this study including them without valid HbA1c data could have diluted or confounded the true relationships between the studied variables. Therefore, we recommend further studies to explore more of the factors that could affect adequate monitoring of glycemic control among this study population.”** | **201 - 207** |
