# Supplementary material for: Prevalence and determinants of poor glycemic control among diabetic chronic kidney disease patients on maintenance hemodialysis in Tanzania
Source: PLoS One. 2025 Feb 10;20(2):e0306357. doi: 10.1371/journal.pone.0306357 (PMC11809847; doi:10.1371/journal.pone.0306357)
Supplement: Attachment — Submitted filename: matrix_response_for DCKD Manuscript.docx [file pone.0306357.s004.docx]

**RESPONSE REVIEWER COMMENTS**

**Title: Prevalence and determinants of poor glycemic control among diabetic chronic kidney disease patients on maintanance hemodialysis in Tanzania**

| **S/N** | **Comments from the reviewer** | **PI’s response** | **Line Number**  **where changes have been made** |
| --- | --- | --- | --- |
|  | **Journal Requirements:**  Please review your reference list to ensure that it is complete and correct. If you have cited papers that have been retracted, please include the rationale for doing so in the manuscript text, or remove these references and replace them with relevant current references. Any changes to the reference list should be mentioned in the rebuttal letter that accompanies your revised manuscript. If you need to cite a retracted article, indicate the article’s retracted status in the References list and also include a citation and full reference for the retraction notice. | The list has been reviewed for completeness and we confirm that all references are correct.  Changes have been on citation of reference 9, 14 and 21. While the links to the references 14 and 21 have been added, reference number 9 is a local guideline available only in the printed form and is not available online. | Page 17 and 18 |
