# Supplementary material for: Prevalence and determinants of poor glycemic control among diabetic chronic kidney disease patients on maintenance hemodialysis in Tanzania
Source: PLoS One. 2025 Feb 10;20(2):e0306357. doi: 10.1371/journal.pone.0306357 (PMC11809847; doi:10.1371/journal.pone.0306357)
Supplement: Attachment — Submitted filename: matrix_response_for DCKD Manuscript (2).docx [file pone.0306357.s005.docx]

**RESPONSE REVIEWER COMMENTS**

**Title: Prevalence and determinants of poor glycemic control among diabetic chronic kidney disease patients on maintanance hemodialysis in Tanzania**

| **S/N** | **Comments from the reviewer** | **PI’s response** | **Line Number**  **where changes have been made** |
| --- | --- | --- | --- |
|  | Please note that funding information should not appear in the Acknowledgments section/Funding section or Any other areas of your Manuscript. We will only publish funding information present in the Funding Statement section of the online submission form. Please remove any funding-related text from the manuscript. | Funding section has been deleted. | Page 16 |
